# The Role of Interruptions in polyQ in the Pathology of SCA1

**DOI:** 10.1371/journal.pgen.1003648

**Published:** 2013-07-25

**Authors:** Rajesh P. Menon, Suran Nethisinghe, Serena Faggiano, Tommaso Vannocci, Human Rezaei, Sally Pemble, Mary G. Sweeney, Nicholas W. Wood, Mary B. Davis, Annalisa Pastore, Paola Giunti

**Affiliations:** 1 MRC NIMR, London, United Kingdom; 2 Department of Molecular Neuroscience, UCL Institute of Neurology, London, United Kingdom; 3 Department of Protein Macroassemblies and Prion Pathology, INRA, Domain de Vilvert, Jouy en Josas, France; 4 Neurogenetics Unit, National Hospital for Neurology and Neurosurgery, London, United Kingdom; The Hospital for Sick Children and University of Toronto, Canada

## Abstract

At least nine dominant neurodegenerative diseases are caused by expansion of CAG repeats in coding regions of specific genes that result in abnormal elongation of polyglutamine (polyQ) tracts in the corresponding gene products. When above a threshold that is specific for each disease the expanded polyQ repeats promote protein aggregation, misfolding and neuronal cell death. The length of the polyQ tract inversely correlates with the age at disease onset. It has been observed that interruption of the CAG tract by silent (CAA) or missense (CAT) mutations may strongly modulate the effect of the expansion and delay the onset age. We have carried out an extensive study in which we have complemented DNA sequence determination with cellular and biophysical models. By sequencing cloned normal and expanded SCA1 alleles taken from our cohort of ataxia patients we have determined sequence variations not detected by allele sizing and observed for the first time that repeat instability can occur even in the presence of CAG interruptions. We show that histidine interrupted pathogenic alleles occur with relatively high frequency (11%) and that the age at onset inversely correlates linearly with the longer uninterrupted CAG stretch. This could be reproduced in a cellular model to support the hypothesis of a linear behaviour of polyQ. We clarified by *in vitro* studies the mechanism by which polyQ interruption slows down aggregation. Our study contributes to the understanding of the role of polyQ interruption in the SCA1 phenotype with regards to age at disease onset, prognosis and transmission.

## Author Summary

Spinocerebellar ataxia type 1 (SCA1) is a progressive neurodegenerative disorder resulting in loss of coordination and balance. It is caused by an expanded repeated DNA sequence (CAG) in the gene *ATXN1*. The CAG repeat region is normally interrupted by the DNA sequence CAT. Loss of this interruption is believed to cause instability whereby the CAG repeat expands beyond a key threshold resulting, ultimately, in polyglutamine protein aggregation and cell death. Here we examine how interruptions influence pathology in patients and establish a cellular model to support our findings. We distinguish our patients into two sub-groups based on whether or not their expanded CAG repeat stretches contained an interruption. This is not possible with conventional diagnostic techniques. Differentiating the sub-group with no interruptions led to improved accuracy in predicting their age at onset. The other sub-group, with interruptions, reveals a delay in age at onset that shows greater alignment with the longest stretch of CAG repeats. These findings are significant for genetic counselling and prognosis. Our cellular model and *in vitro* studies confirmed the relationship between disease severity and uninterrupted repeat length and showed that interruptions do not significantly affect the polyglutamine protein aggregation, but do slow down the aggregation rate.

## Introduction

Anomalous expansion of coding CAG repeats in specific genes is the cause of at least nine different neurodegenerative diseases that include Huntington's chorea, Kennedy's Disease and various types of spinocerebellar ataxias [1]. Despite the clinical and genetic heterogeneity of these disorders, a common hallmark of their pathologies is the presence of neuronal intranuclear protein aggregates with a granular or fibrillar morphology in the affected cells, which are strongly reminiscent of those observed in Alzheimer or Parkinson's diseases [2]. Although their role is still debated, increasing evidence indicates that these inclusions and/or their soluble precursors are highly cytotoxic and a direct cause of disease. *In vitro* and *in vivo* studies have demonstrated that polyQ is insoluble and that aggregation depends on the polyQ length and concentration [3], [4]. While polyQ expansion is essential for triggering disease, other regions of the carrier proteins may modulate the aggregation properties and the severity of the pathology.

The age at onset and the severity of the polyQ expansion diseases inversely correlate with the length of the polyQ tract provided that this exceeds a threshold specific for each disease, which is in most cases around 35–40 repeats [5]–[7]. It was initially suggested that the threshold could be explained by different structural features of polyQ when the polyQ length is above or below it [8], [9]. However, neither we nor other groups could collect any evidence in support of different length-dependent structural properties [10]–[17]. We observe instead that polyQ of different lengths have different aggregation kinetics, suggesting that the difference between pathology and health is determined by this property [13].

Cases in which there are silent or missense mutations within the polyQ tract have been observed [18]–[26]. The specific codon composition that encodes polyQ tracts appears to determine the susceptibility of an allele to expansion: while polyQ is also encoded by the CAA codon, the polyQ tracts observed in disease-causing genes seem to being the majority of the cases composed of long uninterrupted repeats of CAG triplets. Interestingly, polyQ encoded by mixtures of CAG and CAA codons seems to be less prone to expansion.

Interruptions strongly modulate the effect of expansion on pathology. For instance, individuals with expanded ataxin-1, the protein responsible for spinocerebellar ataxia type 1 (SCA1), but carrying histidine (CAT) interruptions were reported to be phenotypically normal [27], [28], suggesting that interruptions could alter the polyQ properties and reduce the toxic effects.

Although the presence of interruptions and their influence on pathology has been reported for some time [27]–[31], there is still little evidence that can explain their exact influence on the SCA pathologies. It is known that interruption of the expanded allele gives more stability during transmission, independently of the sex of the transmitting parent. The converse applies when the CAG tract of the expanded allele is pure [32]–[34]. Therefore, knowing whether an interruption is present is crucial in genetic counselling of patients, particularly with respect to prediction of age at onset and progression of the disease as well as to the probability of allele expansion when transmitted to future generations. This information could also guide the design of future effective therapeutic strategies.

In this study we present a detailed investigation aimed at further understanding the role of polyQ interruptions. We carried out an extensive genetic study on 36 individuals to understand the sequence and length variations of the repeat region in both alleles due to mosaicism. We also analysed parent-child transmission in two families. The first family has a pure CAG repeat in their expanded allele. As expected from previous studies [32], [34], the transmission from the mother led to a contraction of the repeat tract in her son, whilst the allele underwent expansion on transmission from her son to her grandson. The second family has an interrupted CAG repeat tract in their expanded allele. Surprisingly, maternal transmission of the expanded allele in this family led not only to the loss of the interruption, but also to the contraction of the pure CAG repeat tract in the expanded allele. Our work strongly suggests the importance of directly assessing the expanded CAG repeat allele in diagnosis. We show for the first time that alleles with interruptions are not necessarily stable. This is at variance with current diagnostic tests for SCA1 which rely on fragment sizing SCA1 alleles, a method that does not discriminate between interrupted and uninterrupted alleles. We complemented the genetic data by *in vitro* studies both using cellular models and biophysical methods. Our results clarify the mechanism of aggregation and show that the presence of interruptions affects fibre formation stability.

## Results

### SCA1 patient clone analysis

A detailed strategy was devised for cloning the CAG repeat region from alleles of the gene responsible for SCA1 (*ATXN1*) into a plasmid vector, from which the repeat configuration could be determined by sequencing. Cloning rather than direct sequencing of the CAG repeat region was necessary because the mosaicism caused by the repeats results in a mixed population of repeat lengths [21], [32]. A high-fidelity proof-reading DNA polymerase (Phusion) was used to minimise potential errors when amplifying the CAG repeat region from patient DNA. PCR primers used for amplifying the CAG repeat region for *ATXN1* were designed to incorporate *Bam*HI and *Xho*I restriction sites for cloning into a pcDNA3.1+ plasmid vector. Pathogenic and normal alleles for each patient were gel-purified and cloned individually into the plasmid vector. To avoid point-mutations from DNA exposure to UV during gel-purification, a counterion-dye DNA staining method with crystal violet and methyl orange was used to post-stain gel-purification gels and visualise DNA by normal white light. Initial experiments involved the cloning of the pathogenic allele for SCA1 patient #1 using the standard *E. coli* cloning strain DH5α and a strain, Stbl3, where the rate of homologous recombination is reduced so direct repeats should be stably replicated. When cloned into DH5α, the pattern of repeats/interruptions varied significantly with interruption patterns that could only be explained by artefacts of homologous recombination in this strain. The sequence of the repeat region was more stable in Stbl3 *E. coli*. This strain was therefore used for subsequent cloning experiments. For the majority of alleles cloned, a minimum of five colonies from the bacterial transformation plate were analysed to determine a range of the individual repeat sequences. Normal and pathogenic alleles of 36 individuals (35 SCA1 patients and one SCA3 patient with a borderline SCA1 expansion) were cloned and sequenced. A total of 800 clones were generated from these patients, comprising 101 unique repeat region clones (40 uninterrupted and 61 interrupted CAG repeat regions, see **Table S1**).

Since the number of clones sequenced for each individual differed, clone numbers were expressed as a percentage to account for clone depth (see **Table S2)**. For all patients we observed a diverse distribution of lengths both for the normal and the expanded allele with some patterns being significantly more represented than others. The majority of uninterrupted clones (93%) had an allele size above the pathogenic threshold of 39 repeats or greater, whilst the majority of interrupted clones (90%) had an allele size below the pathogenic threshold. The most frequent uninterrupted allele sequence (CAG)_47_ was seen in 5% of all clones whilst the most frequent interrupted allele sequences were [(CAG)_12_(CAT)(CAG)(CAT)(CAG)_14_] and [(CAG)_12_(CAT)(CAG)(CAT)(CAG)_15_], comprising 16% and 14% of all clones, respectively. Interruptions were observed in both normal and pathogenic alleles. For 88% of clones with interrupted sequences, the interruption pattern was [(CAT)(CAG)(CAT)] while a single CAT interruption was observed in 10% of interrupted clones. Most of the remaining interrupted clones had three CAT interruptions in the pattern [(CAT)(CAG)(CAT)(CAG)(CAT)] whilst a single case had four CAT interruptions in the pattern [(CAT)(CAG)(CAT)(CAG)(CAT)(CAG)(CAT)]. Patient 9 had a single clone (out of 19) with a novel single AAG interruption. This is however probably due to a PCR or sequencing artefact.

Finally, the most important observation arising from these data is that we have different examples of interrupted expanded pathogenic alleles in which uninterrupted regions are well above the pathological threshold. Such an observation has been reported only once before for a SCA1 patient with an interrupted expanded pathogenic allele of a total length of 58 repeats and with 45 repeats in the longest uninterrupted stretch [35]. However, in this reported case the total number of repeats in the interrupted allele and the longest uninterrupted CAG stretch was much shorter than the cases presented here. For instance, patient #3 had a single clone of total length 83 repeats with the sequence [(CAG)_70_(CAT)(CAG)(CAT)(CAG)_10_] whilst patient #1 had multiple clones of total length ranging in size from 64–69 repeats with the sequences [(CAG)_51–56_(CAT)(CAG)(CAT)(CAG)_10_]. To our knowledge, these are the longest interrupted pathogenic alleles reported to date.

### Influence on the age at disease onset of polyQ interruptions in the pathogenic allele

**Figure 1:**
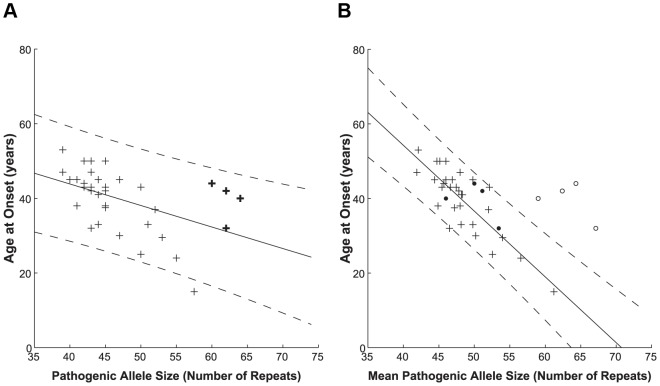
Correlation between polyQ length expansion and age at disease onset. Pathogenic allele size as determined via diagnostic sizing (**A**, *n* = 35) and by clone sequencing (**B**, *n* = 35). Interrupted alleles are indicated on the diagnostic sizing graph by bold crosses for reference only, since these cannot be differentiated by this method. The clone sequencing approach further sub-divided the patient group into those with primarily uninterrupted (crosses, *n* = 31) and interrupted (open circles – total allele length; filled circles – longest CAG stretch, *n* = 4) allele repeats; these subsets are not distinguished via conventional sizing. A linear model was used to relate the patient's age at disease onset to their indistinguishable pathogenic allele (**A**) or uninterrupted pathogenic allele (**B**) size. The solid lines depict the fit result while the dashed lines show the prediction bounds at the 95% confidence level. Sequencing of cloned patient alleles produces a higher quality fit with tighter prediction bounds. The data used to prepare this figure may be found in **Table S3**.

Having this unprecedented number of sequences, we checked the dependence of age at onset on the repeat lengths and compared our results with those obtained by fragment sizing, the method commonly used in diagnostic laboratories. This method does not allow for a differentiation of interrupted and uninterrupted pathogenic alleles, since the supplemental step of *Sfa*NI digestion to detect CAT interruptions is not routinely performed on large pathogenic alleles. When plotting the age at disease onset against the pathogenic allele size as determined by fragment sizing (**Figure 1A**) we found a correlation with a Pearson correlation coefficient *r* = −0.490 (significant at the 0.01 level). Such correlation is in keeping with previous studies [20], [34], however the quality of fit to the linear model is low (A = −0.577, b = 66.910, adjusted R^2^ = 0.212).

The clone sequencing approach allows patients to be sub-divided based on whether or not they have an interrupted pathogenic allele. For the purposes of this study, the allele size for interrupted alleles is the total length of the polyQ tract, including CAT interruptions. When we plot the age at disease onset against mean uninterrupted pathogenic allele size as determined by clone sequencing (**Figure 1B**, crosses) we found a significant correlation to the pathogenic allele size, having a Pearson correlation coefficient *r* = −0.825 (significant at the 0.01 level). The quality of fit to the linear model greatly increases (A = −1.764, b = 124.817, adjusted R^2^ = 0.670) as compared to the fragment sizing data (0.670 compared to 0.212). This indicates that this model describes the data better and therefore the clone-sequencing data predict much more accurately the age at disease onset. The steeper slope determined by the clone-sequencing derived fit also indicates a more sensitive determination of the age at disease onset from the pathogenic allele size. As in Jodice *et al.* (1994) [20], [34], our data showed a slightly better fit to the linear model compared to the exponential model (adjusted R^2^ = 0.670 compared to 0.641, data not shown), probably due to the lack of subjects with extremely early or late ages at disease onset.

Interrupted pathogenic alleles (**Figure 1B**, open circles) form a distinct cluster outside the prediction bounds at the 95% confidence level of the uninterrupted pathogenic allele linear model. Interruption of the pathogenic allele is known to delay age at onset in agreement with the shift to a later age at onset observed here. If instead the mean longest contiguous stretch of CAG repeats is plotted against age at disease onset (**Figure 1B**, filled circles), these points shift to fall within the linear model for the uninterrupted pathogenic alleles.

To determine whether there is a statistical significance in the difference between the fragment sizing and sequencing models we compared the magnitude of the residuals, i.e. the difference between the age at onset data and that predicted by each model. A two sample, one-tailed *t*-test was performed with the null hypothesis (H_0_) that the mean of the absolute residuals of the sizing fit was not greater than those of the sequencing fit. The variances were not assumed to be equal following a Levene's test (*P* = 0.016). The null hypothesis could be rejected with *P* = 0.041.

Most of our clones were also analysed by diagnostic fragment sizing. We see very little difference between repeat sizes from both methods (**Figure S1**). The sizing of the clones by the two methods are very significantly correlated with a Pearson correlation coefficient *r* = 0.999 (significant at the 0.01 level) and high quality of fit to the linear model (A = 0.952, b = −0.822, adjusted R^2^ = 0.999). The maximum difference between the two methods was about 5 repeats. This is in keeping with the repeat differences between pathogenic alleles that were diagnostically fragment sized from patient DNA and the mean size from sequenced clones.

Our studies indicate the importance of clone-sequencing in improving the estimation of age at disease onset, which must be estimated from the longest uninterrupted CAG stretch rather than from the total length of polyQ when these are interrupted.

### Patients with interrupted CAG repeat tracts in their expanded alleles

When interpreting the prognosis for patients, knowing the repeat pattern appears to be particularly critical. Our analysis shows that interruption in the expanded allele seems to markedly delay the age at disease onset. Subject #1 was of particular interest as this individual had an allele with the pattern (CAG)_51–56_(CAT)(CAG)(CAT)(CAG)_10_. Despite the interruptions, the length of the uninterrupted 5′ stretch is well above the threshold. The patient was initially examined when she was 46 years old. Both her mother and a sibling were affected by SCA1. Using the linear model for sequenced uninterrupted pathogenic alleles described here, one would predict an age at disease onset of 16 years old ±12 years for this patient's total allele repeat size (indeed, individual #17 had a similar sized uninterrupted repeat with an age at onset of 15 years), so one would expect the patient to have already developed the disease. The patient instead had a history of only four years of gait disturbance and numbness in her legs. Ataxia was affecting her four limbs and she had dysarthria, poor sleep and impaired swallowing. Her ankle reflexes were absent and plantar responses were extensor. The patient was able to walk independently, revealing that disease progression was slower than expected given the total number of CAG repeats. No sensory abnormalities were detected.

A second patient (subject #4) had a similar interrupted pathogenic allele pattern (CAG)_50–53_(CAT)(CAG)(CAT)(CAG)_9–10_. Once again the length of the uninterrupted 5′ stretch is above the pathogenic threshold. This patient had an age at onset of 44 years old and died 23 years later. Both of her daughters (subjects #5 and #6) were also diagnosed with SCA1, having an age at onset of 41 and 43 years old, respectively. However, their pathogenic allele appears to have lost the interruption. This family is further discussed later in the Parent-Child Analysis section.

Subject #3 was the third patient with an interrupted pathogenic allele. Clones were identified with sequence pattern of (CAG)_52–58_(CAT)(CAG)(CAT)(CAG)_9–10_ and (CAG)_70_(CAT)(CAG)(CAT)(CAG)_10_. One clone was also identified with the sequence pattern, (CAG)_4_(CAT)(CAG)_6_(CAT)(CAG)_40_(CAT)(CAG)(CAT)(CAG)_10_. Each of these alleles contains a contiguous stretch of CAGs above the pathogenic threshold. The age at disease onset for this patient was 32 years old.

A fourth patient (subject #2) also has an interrupted pathogenic allele with a pattern (CAG)_46_(CAT)(CAG)(CAT)(CAG)_10_. The length of the uninterrupted 5′ stretch is above the pathogenic threshold and her age at onset was about 40 years old.

Subject #36 had a SCA1 allele fragment sized at 39 repeats, which could be digested with *Sfa*NI and therefore she was not given a diagnosis of SCA1. Further investigation showed that the patient had polyQ expansion in *ATXN3* (69 repeats). This patient had SCA1 alleles with the interruption pattern (CAG)_19–20_(CAT)(CAG)(CAT)(CAG)_17–19_, confirming that she did not have SCA1.This case supports our confidence in the diagnostic power of the correlation between uninterrupted polyQ and disease.

Three other patients (#7, #8 and #23) each had a single clone with interruptions. Comparison with other patients with the same age at onset revealed a similar mean CAG repeat size. Indeed, these patients are indistinguishable from patients with pure uninterrupted alleles of the same size (see **Figure S2**, where these three patients are highlighted in red). This suggests having only one interrupted clone does not have an effect on the phenotype. Since these patients only have a single interrupted clone within a population of uninterrupted clones these interruptions could have arisen from a PCR artefact. A possible source of artefacts could be the formation of chimeric PCR products, as previously reported [36]. If there is incomplete extension of the DNA during the PCR extension step, the PCR product can act as a primer in subsequent rounds of replication. Template switching may then occur between normal and pathogenic alleles, introducing interruptions in the (CAT)(CAG)(CAT) configuration to the PCR product.

### Parent-child analysis

**Figure 2:**
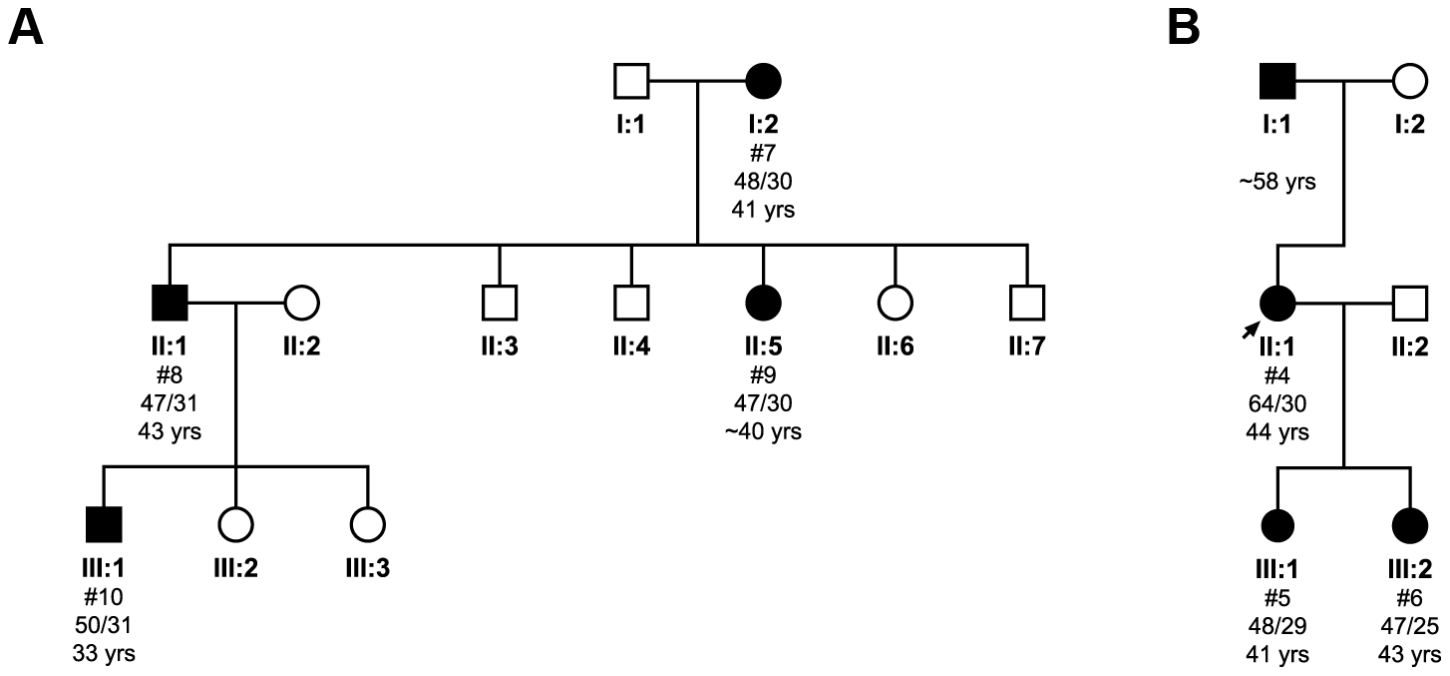
Pedigree analysis and the transmission of the pathogenic expanded allele in two families. Subject numbers (#) correspond to cloned individuals indicated in **Table S3**, where individual cloned sequences can be found. The mean total repeat size as determined by clone sequencing for each allele is shown, along with the age at disease onset. The first family (**A**) illustrates the transmission of a pure CAG repeat tract across three generations. The second family (**B**) shows transmission of an interrupted pathogenic allele. The limited number of clones analysed may have introduced some degree of sampling error.

In the first pedigree (**Figure 2A**), the transmission of the primarily uninterrupted CAG repeat tract expanded allele was monitored through three generations, with both maternal and paternal transmission occurring. The expanded allele appears to contract from an average of (CAG)_48_ to (CAG)_47_ when passed from mother (I:2) to son (II:1), and daughter (II:5). Expansion from (CAG)_47_ to (CAG)_50_ occurs when transmitted from son (II:1) to grandson (III:1). Correspondingly, there is a slight delay in the age at disease onset of two years between mother and son (41 and 43 years old, respectively), whilst the grandson had an age at disease onset ten years earlier than his father (33 years old). Interestingly, the mother and son had 44 repeats when sized diagnostically, whilst cloning and sequencing identified a contraction from a mean value of 48 to 47 repeats. This difference could however be mediated by sampling error due to the small number of clones analysed for these patients.

The second pedigree (**Figure 2B**) shows transmission of an interrupted CAG repeat tract expanded allele. The proband (II:1) has a repeat tract pattern of (CAG)_50–53_(CAT)(CAG)(CAT)(CAG)_9–10_, which on transmission to one daughter (III:1) contracts to an uninterrupted (CAG)_48_ repeat tract allele and to the other (III:2) contracts to an uninterrupted (CAG)_47_ repeat tract allele. The PCR products of both daughters are resistant to *Sfa*NI digestion (data not shown), confirming uninterrupted expanded alleles. Despite the contraction of the repeat, we also see a loss of the interruption, which led to an earlier age at onset of 41 years in one daughter (III:1) and 43.5 year in the other daughter (III:2), as compared to 44 years in the proband. The proband inherited her expanded allele from her father, who, consistent with paternal transmission, had an age at onset in his late-fifties, several years later than the proband.

### His insertion in expanded polyQ reduces aggregate formation in transfected cells

To further understand the role of interruptions in ataxin-1, the protein responsible for SCA1, we developed a cellular model system in which we could transiently transfect constructs with interrupted and non-interrupted polyQ tracts to see how interruption, the specific pattern and the total length would affect aggregate formation. Such a cellular model, albeit simple, has proven powerful for studying the effect of polyQ in cell [37]–[40]. We used ataxin-1 constructs in which the C-terminus of the protein up to the polyQ stretch (ataxinCT) was fused to various synthetic polyQ or polyQH (with histidine interruptions) stretches. Truncation of about 200 N-terminal amino acids was dictated by technical reasons since we could otherwise not easily insert the wanted stretch into the full-length protein. The excluded N-terminus has no known role in aggregation or other function, whereas what we retained spans most of the protein and includes the C-terminus where the functional motifs involved in aggregation and toxicity are located, such as the AXH domain, the phospho-serine 776 and the nuclear localization signals [37], [41]–[45].

We selected different interruption patterns (**Table 1**): interrupted patterns of three different total lengths (30Q, 54Q and 82Q) were compared with corresponding uninterrupted polyQ stretches. The effect of different interruption patterns in the expanded range was assessed with four sequences of similar total length (64-69Q), two of which (65Q and 69Q) were taken from the patterns observed for patient #1.

**Table 1:** Aggregation tendency indicated by cell counting analysis.

PolyQ Nr	%	Repeat pattern with His interruptions	%
Q_30_	7.8±0.4	(Q)_10_(H)(Q)_7_(H)(Q)_11_	7.3±0.7
Q_54_	28.5±1.0	(Q)_30_(H)(Q)_11_(H)(Q)_11_	17.1±0.8
Q_64_	34.7±1.5	(Q)_50_(H)(Q)_6_(H)(Q)_6_	27.8±0.6
Q_64_	34.7±1.5	(Q)_11_(H)(Q)(H)(Q)_16_(H)(Q)(H)(Q)_18_(H)(Q)(H)(Q)_10_	11.3±1.2
Q_65_	34.9±1.4	(Q)_52_(H)(Q)(H)(Q)_10_*	29.4±0.7*
Q_69_	35.4±1.1	(Q)_56_(H)(Q)(H)(Q)_10_*	30.3±0.9*
Q_82_	58.2±2.5	(Q)_30_(H)(Q)_12_(H)(Q)_12_(H)(Q)_12_(H)Q_12_	21.3±1.7

COS cells were transfected with various ataxinCT constructs fused with polyQ repeats or repeats containing His interruptions. 48 h after transfection, cells were stained and analysed by fluorescent microscopy and the percentage of cells with aggregates was determined. Approximately three hundred cells were counted in each experiment. Data represent means ± SD of at least three experiments. Asterisks indicate sequence patterns corresponding to those identified in patient samples.

**Figure 3:**
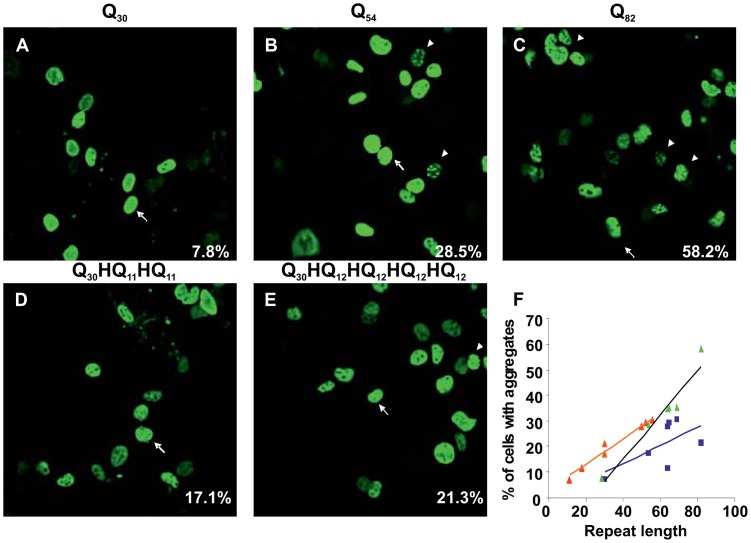
Interruption of the expanded polyQ stretch by histidines reduces its aggregation in transfected cells. COS cells were transfected with uninterrupted (**A, B and C**) or His interrupted polyQ-ataxinCT constructs (**D and E**). The repeat pattern is shown above each panel. Cells were stained with anti-ataxin-1 antibodies and FITC conjugated secondary antibodies. The % of cells with aggregates (average from at least 3 different experiments) is shown at bottom right hand corner of each panel. Arrows indicate cells with diffused expression of ataxinCT proteins and arrowheads indicate aggregates. (**F**) Analysis of the data in **Table 1** plotting the percentage of aggregates observed in cells expressing uninterrupted polyQ proteins (green triangles, R^2^ = 0.94) and His-interrupted polyQ proteins plotted either against total repeat length (blue squares, R^2^ = 0.40) or against the longest polyQ tract length (orange triangles, R^2^ = 0.97). The regression lines and the R^2^ values were calculated using Microsoft Excel.

Uninterrupted polyQ-ataxinCT formed aggregates (**Figure 3A–C**) in a manner comparable to that previously observed for full-length ataxin-1 [37]. When the cells were transfected with interrupted polyQ-ataxinCT, we observed a clear reduction of the number of cells containing aggregates (**Figure 3D,E**). Semi-quantification of the effect by estimating the percentage of cells containing aggregates (**Table 1**) shows a clear difference between uninterrupted and interrupted sequences. A plot of uninterrupted polyQ length versus the percentage of cells with aggregates shows an excellent linear correlation (**Figure 3F**) (R^2^ = 0.94). Interestingly, when the same plot is obtained for interrupted polyQ stretches optimal correlation is obtained only when using the longer stretch of uninterrupted polyQ, independently of the pattern (R^2^ = 0.97 versus 0.40 when using the total polyQ length). The effect is particularly evident when comparing the different patterns with the same or approximately the same total length: only 11% of the cells transfected with the polyQ pattern Q_11_HQHQ_16_HQHQ_18_HQHQ_10_, contain aggregates as compared to 28% in those transfected with Q_52_HQHQ_10_ and 29% in those transfected with Q_56_HQHQ_10_.

These results indicate that the observed correlation directly reflects a different behaviour between interrupted and uninterrupted polyQ and that, although very simple, our model faithfully reproduces the correlation between length and disease onset age observed in patients

### His interruptions reduce formation of SDS-insoluble aggregates in transfected cells

To test the aggregate stability for different polyQ patterns, we carried out filter retardation assays on the pellet of the transfected cells. These assays exploit the observation that SDS-insoluble protein aggregates from cells expressing expanded polyQ fusion proteins are retained on a cellulose acetate filter [46], [47]. Formation of these high molecular weight aggregates was shown to occur in a repeat length dependent manner [46], [47].

**Figure 4:**
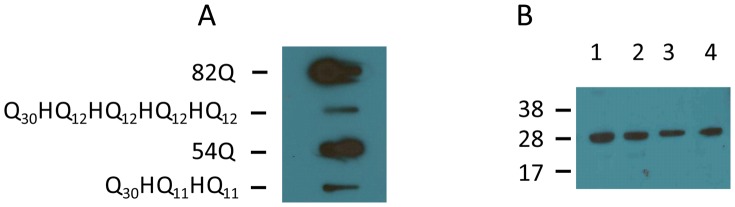
Detection of protein aggregates formed in transfected COS cells by slot blot filter assay. Slot blot filter assay was carried out on the insoluble fraction isolated from transfected COS cells. COS cells were transfected with ataxinCT constructs expressing polyQ repeats with and without His interruptions. Cell pellets obtained after lysis were treated with DNAse I, boiled in 2% SDS and filtered through a cellulose acetate membrane. Aggregated protein retained on the membrane was detected with anti-ataxin-1 antibodies. (**A**) Proteins retained from uninterrupted (82Q; 54Q) and interrupted (Q30HQ12HQ12HQ12HQ12; Q30HQ11HQ11) polyQ expressing cells. Results were consistent across three separate experiments. (**B**) Supernatants obtained after cell lysis from each of the above samples were subjected to immunoprecipitation using anti GFP antibodies and the precipitates were subjected to western blot analysis using anti-GFP antibodies. Lane 1, uninterrupted Q82; lane2, interrupted 82Q; lane 3, uninterrupted 54Q; lane 4, interrupted 54Q. Molecular weight markers are indicated on the left.

After transfecting COS cells with appropriate plasmid vectors carrying His interrupted or uninterrupted polyQ tracts, cell lysates were prepared. The insoluble material was collected by centrifugation and treated with DNase I. The resulting protein mixture was subjected to filter retardation assays. Insoluble protein aggregates were found to form when cells were expressing 54 or 82 repeats (**Figure 4A**, 82Q and 54Q). Cells expressing His interrupted polyQ of the equivalent length also contained insoluble protein aggregates (**Figure 4A**, Q_30_HQ_12_HQ_12_HQ_12_HQ_12_and Q_30_HQ_11_HQ_11_). In both cases the band intensity of interrupted polyQ was lighter than that from cells expressing uninterrupted polyQ proteins.

To ensure that the observed difference is not caused by marked differences in expression levels of interrupted and polyQ expanded constructs, supernatants obtained after cell lysis from each of the above samples were immunoprecipitated using antibodies against the internal transfection control and expression levels were assessed by western blotting (**Figure 4B**). The resulting blots show similar protein contents. The transfection control GFP was not detectable in the SDS insoluble ataxin-1 containing protein aggregates (not shown), indicating that the expressed GFP remained in the soluble part of the lysates.

Taken together, these data confirm a clear difference between interrupted and uninterrupted polyQ sequences that could be explained either by a reduced aggregate formation tendency or by a lower stability of the aggregates. This is in agreement with earlier studies on the effect of interruptions using GST fused polyQ proteins expressed in E. coli [30].

### His interruptions of polyQ decrease fibre formation rates and increase oligomer stability

To further characterize the aggregation mechanism of interrupted polyQ, we used polyQ peptides fused to GST. This *ad hoc in vitro* model has proven a powerful alternative tool to solid phase chemical synthesis [3], [13], [14], [46], [47]. This system offers the advantage that polyQ can be rapidly cleaved off from GST by proteolysis (the reaction with thrombin is complete in 5 minutes at 0.5 U of enzyme per nmole of GST protein), allowing the study of polyQ having it in a predominantly monomeric state at time zero. Fusion with the highly soluble GST allows recombinant expression and purification of polyQ chains up to about 40–45 repeats and reduces their tendency to aggregate [14], [30], [48]. Beyond this length the solubilising effect of GST becomes weaker so that it is hard to obtain monodispersed samples (Masino and Pastore, unpublished observations). We produced two constructs, one with an uninterrupted polyQ stretch (polyQ41) and one with a Q_22_HQ_19_ pattern (polyQ41H). After cleavage the polyQ peptides contain only two non-native residues (GS).

**Figure 5:**
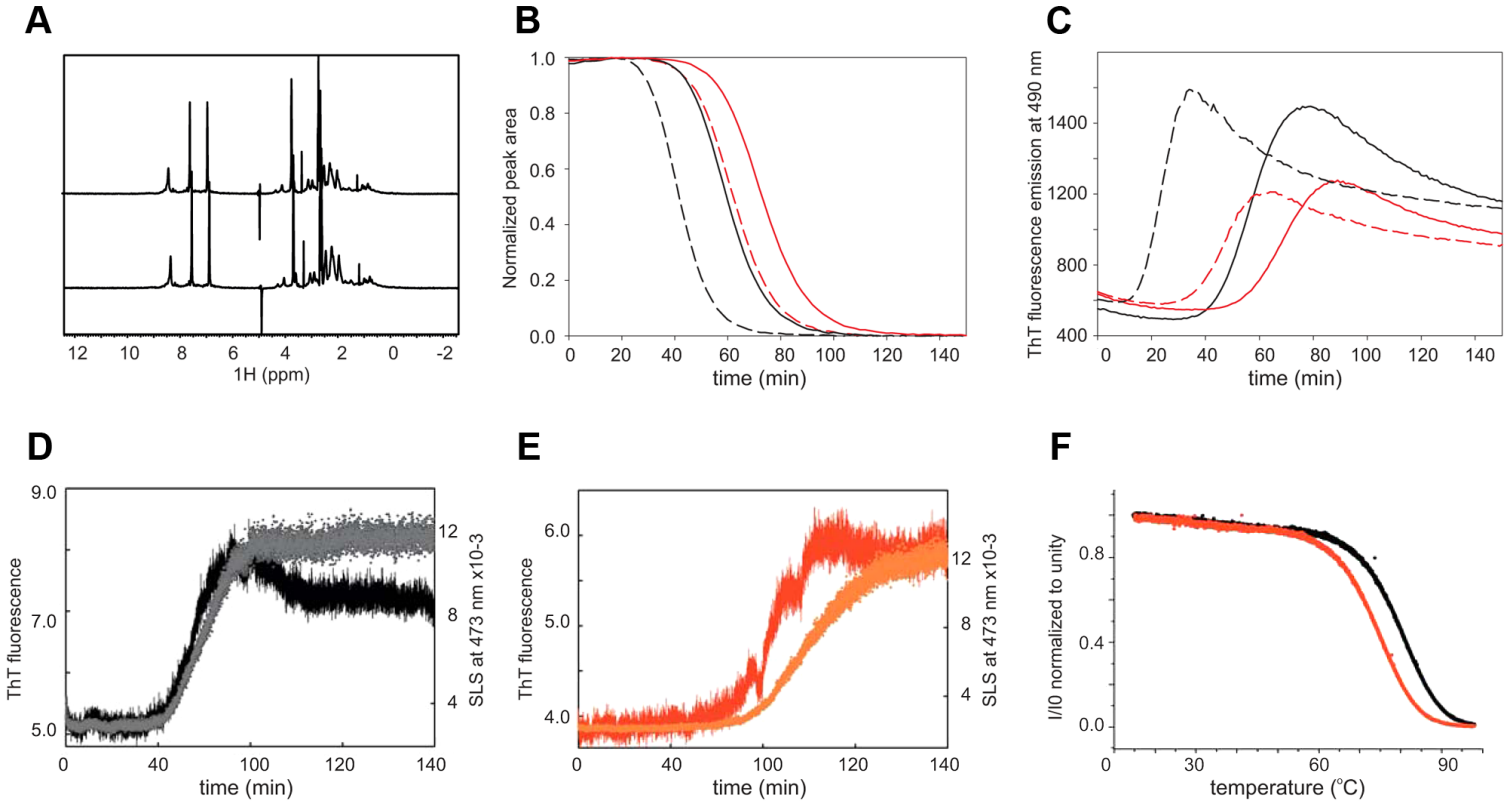
Comparison of the structural and aggregation properties of interrupted and uninterrupted polyQ. (**A**) ^1^H NMR spectra of interrupted (upper trace) and uninterrupted (lower trace) of the GST fused polyQ41 and polyQ41H peptides recorded at 600 MHz and 10°C on 50 µM protein samples in 50 mM sodium phosphate at pH 6.9 and 3 mM DTT. (**B**) Aggregation kinetics followed by NMR collected forpolyQ41 (black) and polyQ41H (red). Continuous and broken lines are used for filtered and non-filtered samples respectively. (**C**) Aggregation kinetics obtained by ThT fluorescence detection at 25°C in a stirring plate reader. The conventions are the same as in (**B**). (**D**) Simultaneous recording of the SLS, plotting the absorbance at 473 nm (black), coupled with ThT fluorescence aggregation kinetics (grey) for polyQ41. (**E**) The same as in (**D**) for polyQ41H (in red and orange respectively). The measurements were carried out at 37°C. (**F**) Plot of the light scattering intensity as a function of temperature for polyQ41 (black) and polyQ41H (red).

We first checked by nuclear magnetic resonance (NMR) if we could detect differences in the structure of interrupted and uninterrupted polyQ in solution. Comparison of the one-dimensional ^1^H spectra of the uncleaved proteins (50 µM samples in 50 mM phosphate at pH 6.9, and 3 mM DTT) shows that they have very similar features (**Figure 5A**). The intense peaks at 8.2, 7.4 and 6.8 ppm correspond to the backbone and side chain amides of polyQ that are indicative of a flexible random coil conformation. Upon thrombin cleavage, the spectra remain invariant except for the progressive disappearance of the polyQ signals as a consequence of the formation of high molecular weight aggregates that are too large to be detectable by liquid state NMR.

We followed the kinetics of aggregation by monitoring the rates of disappearance of the polyQ resonances acquiring one-dimensional ^1^H NMR spectra every 5 minutes (**Figure 5B**). The signal decay both at 10 and 25°C is significantly slower for the polyQ41H construct than that of polyQ41 (data not shown). The interrupted polyQ peptide aggregates with a longer lag phase as compared to the uninterrupted polyQ41 peptide. To appreciate the effect of seeding we repeated the experiments after pre-filtering the samples. We observed that aggregation is slowed down but the difference between interrupted and uninterrupted samples is retained indicating that the difference is genuine and is not caused by different seeding effects. The trend was confirmed by Thioflavin T (ThT) fluorescence assays that are routinely taken as a proof for aggregation proceeding through amyloid rather amorphous formation [49], [50]. Also in this case the kinetics of polyQ41H is slower independently of the pre-treatment (**Figure 5C**). Overall polyQ41H has a lower fluorescence signal suggesting that the interruption makes polyQ less susceptible to ThT staining. These data are well in agreement with a previous study carried out on polyQ of non-pathological length (polyQ30) interrupted and uninterrupted peptides [10]–[17].

To compare more directly the kinetics of aggregation and fibre formation of the samples we used a classical microplate reader which allows simultaneous measurement of static light scattering (SLS) and ThT fluorescence in the same cuvette. In SLS measurements, the intensity of the scattered light is directly proportional to the average molecular weight of the particles (i.e. aggregates) in solution. The ThT signal confirms that polyQ41 aggregates faster than polyQ41H (**Figure 5D,E**) and SLS kinetics follows the same behaviour. However, the time scales of the ThT and SLS signals are synchronous only for the uninterrupted polyQ41 whereas aggregation and fibre formations are asynchronous for the interrupted peptide. This observation suggests that amyloid formation is less favoured for the latter leading to a more stable oligomeric state and possibly an internal structural rearrangement leading to ThT binding but with a delayed increase of scattering intensity.

Finally, we wondered whether we could distinguish between the hypotheses that the observed behaviour is caused by a reduced aggregate formation tendency or by a lower stability of the aggregates in interrupted polyQ. We measured the aggregate stability by following the light scattering intensity as a function of temperature. We observed a clear shift (ca. 3°C) of the melting temperature for interrupted polyQ41H that indicates a lower stability.

Taken together, these observations indicate that interruptions do not affect the structure of monomeric polyQ but rather play a role in the kinetics of oligomer and fibre formation.

## Discussion

In this paper, we have presented an extensive characterization of the frequency, behaviour and mechanism of aggregation of interrupted polyQ in SCA1. We studied 36 individuals comprising 35 SCA1 patients and one patient with a borderline SCA1 expansion who was subsequently discovered to have also a SCA3 expansion. To our knowledge this is the largest set of cases in which extensive cloning and sequencing has been carried out for both the normal and expanded alleles. Current diagnostic tests for SCA1 rely on fragment sizing SCA1 alleles, which does not discriminate between interrupted and uninterrupted alleles. When alleles with 35–39 repeats are detected, they are digested with *Sfa*NI to detect CAT interruptions and distinguish large normal and pathogenic alleles. *Sfa*NI digestion is not routinely performed on large pathogenic alleles and therefore interruptions in these alleles are not detected.

We identified 4 out of 35 SCA1 patients (11%) with interrupted pathogenic alleles, a frequency higher than the 1 out of 17 (6%) previously reported [35]. The difference is not statistically significant given the still limited number of samples as also suggested by the Fisher's Exact test with *P* = 0.467. It has been suggested that loss of interruptions predisposes SCA1 (CAG)_n_ to expansion and that they appear only in the normal alleles [32]. This is not substantiated by our findings. We show that interruptions can be common in both alleles and mostly occur through mutation of the last codon position, almost invariably leading to interruption of glutamines by histidines. For both upper and lower alleles, interruptions appear to stabilize the expansion 3′ to the histidine. The CAG repeats seem to be stable 5′ to the interruption for the lower allele with a tendency to have a pattern of 12 CAGs, whereas for the upper allele, the repeat 5′ to the interruption has higher variability and somatic instability in agreement with earlier observations in SCA1 [21], [22], [51] as well as in Huntington's patients [52], [53] and in a Huntington's mouse model [54].

Our findings on one of the families discussed here suggest that the presence of an interruption may influence stability during transmission of the expanded allele (**Figure 2B**). The proband (II:1) has an interrupted pathogenic allele with the longest contiguous CAG stretch containing 50 repeats. Upon transmission to her daughters (III:1 and 2), not only is the interruption lost, but also there is a contraction of the total number of CAG repeats. Although the DNA of the proband's father was not available for analysis, family tree evaluation revealed anticipation with the proband being affected over ten years earlier than her father. This implies that the father may have an interrupted pathogenic allele and expansion may have occurred in the longest contiguous CAG stretch, with the interruption being lost upon subsequent maternal transmission. This is in agreement with one of the mechanisms previously proposed [22], where expansion initially occurs in the CAG tract 5′ to the interruption prior to the loss of the interruption at a later stage leading to a greatly increased instability of the repeat region. This family clearly shows that interrupted repeats can be unstable, and contrasts with the idea of the stability of interrupted repeats. Although unstable interrupted repeats could be rare, the occurrence of instability twice in the present family suggests that it may be a more frequent phenomenon, or that the *ATXN1* gene in this family is particularly prone to instability, or that the mutation from an interrupted to a pure CAG repeat occurred just once in the mother in her postzygotic development and that she is a mosaic with interrupted and uninterrupted alleles. In the latter case it is possible that uninterrupted CAG repeats may be enriched in the proband's oocytes, whilst not being detected in the proband's blood, and therefore be transmitted to her daughters. These findings suggest that the sex of the transmitting parent, even if they have an interrupted pathogenic allele, is important in the diagnosis and prognosis of SCA1.

We have shown that the age at disease onset correlates more significantly with the number of uninterrupted CAG repeats than with the total repeat size including the interruption. Interrupted pathogenic alleles form a distinct cluster outside the 95% confidence level of the uninterrupted pathogenic allele linear model, with a delayed age at onset. If the mean longest uninterrupted stretch of CAG repeats in these interrupted pathogenic alleles is plotted against age at disease onset the correlation falls within the uninterrupted model. This shows that the longest uninterrupted CAG stretch should be considered rather than the total interrupted repeat tract when predicting age at disease onset and pathogenicity. Also this finding has significant implications for patient prognosis and counselling especially if interrupted pathogenic alleles are not identified diagnostically.

We have observed a significant correlation between fragment sizing and sequencing of cloned SCA1 alleles. Interestingly, the larger the repeat size the greater the difference between the fragment sizing and sequencing methods. One explanation for this size-dependent difference between the two techniques is that CAG repeat containing fragments have a high GC-content compared to the commercial size standards used in capillary electrophoresis and hence run through the polymer faster than expected. This is similar to the faster migration of triplet repeats through non-denaturing polyacrylamide gels [55]. The size of these fragments could therefore be interpreted as being smaller than it actually is, as determined by sequencing. Although fragment sizing and sequencing utilise the same polymer (POP-7, Applied Biosystems), the sequence is read from discrete base peaks and does not take into account variations in the migration speed of the fragments, as observed through variations in the breadth of the sequence peaks. The longer the CAG repeat, the more GC-content, which leads to a faster migration and greater difference from the repeat size determined from the repeat sequence. Comparing known sequence clones by fragment sizing has shown that the difference has obvious implications for individuals around the pathogenic boundary. At the pathogenic threshold (≥39 repeats), as defined for the full penetrance pathogenic allele reported in GeneReviews [56], fragment sizing produces repeat values that are about 3 repeats fewer than the known repeat size as determined by sequencing. This suggests that a pathogenic allele of 39 repeats from fragment sizing is actually 42 repeats and that the fragment sizing pathogenic allele threshold should incorporate alleles of at least 36 repeats. Indeed, this is taken into account when fragment sized pathogenic alleles of 35–39 repeats are digested with *Sfa*NI and those alleles that are uninterrupted are deemed potentially pathogenic.

Additional considerations are important for a critical assessment of our results. A total of 800 clones were analysed in this cohort. However, since the number of clones for each patient was relatively low it remains possible that our data may be subject to sampling errors. We have minimised potential PCR errors, but it is not possible to completely eliminate artefacts associated with polymerase slippage and mis-priming [36]. We identified for instance three patients (#7, #8 and #23) who had a single interrupted pathogenic clone within a population of uninterrupted clones. It is likely that these clones are artefacts arising from template switching between normal and pathogenic alleles during PCR as outlined in the Results section. It is also possible that PCR slippage may have affected the lengths of the fragment-sized or sequenced alleles analysed and therefore contributed to the somatic variation observed.

Complementing clinical data with cellular and *in vitro* models has helped us to dissect the mechanism by which histidine interruptions delay the age at onset. Interestingly, we were able to reproduce a similar linear correlation using a cellular model in which we transfected COS cells with C-terminally truncated constructs of ataxin-1 with different polyQ patterns. Also with this model system we observed a satisfactory linear correlation between polyQ length and degree of aggregation only when we considered the longest uninterrupted polyQ stretch. The effect is largely independent from the specific interruption pattern and solely depends on the longer stretch of uninterrupted polyQ.

Our biophysical studies confirm and extend previous work carried out on synthetic polyQ peptides in the non-pathological length range [29], [31]. We provide direct evidence that interruptions do not alter the structure of polyQ which, whether interrupted or not, is in a random coil conformation when in the monomeric state. The aggregation kinetics of interrupted and non-interrupted polyQ are different in a seeding dependent way indicating that interruptions do not affect the final state but alter the kinetic barrier to form aggregates. It was suggested that the differences could be caused either by lower fibre stability or by a lower tendency to aggregate of interrupted polyQ stretches. We are now in the position of discriminating between these two hypotheses. By following a temperature scan with SLS we observed that interrupted polyQ assemblies are less stable. Simultaneous analysis of SLS and ThT fluorescence indicated that the increase in the molecular size and ThT binding occur in a synchronous way for uninterrupted but not for interrupted polyQ for which the increase of the molecular weight occurs after the acquisition of ThT fluorescence. A way to explain these data is by assuming that interrupted polyQ become ThT fluorescent positive before being able to aggregate because they require a structural rearrangement that has a higher barrier than in uninterrupted polyQ.

In conclusion, we have provided an extensive study of the occurrence, significance and mechanism of aggregation of polyQ interruptions that covers different aspects from the genetic to the biophysical characterization. An important consequence of our findings is that all pathogenic alleles should be digested with *Sfa*NI to detect alleles that contain interruptions. This is at variance with current fragment sizing methods for diagnosis that do not routinely differentiate interrupted and uninterrupted pathogenic alleles. Direct sequencing of the expanded allele ultimately provides the only basis for accurately determining the patient's prognosis and age at onset.

## Materials and Methods

### Ethical statement

This research has been approved by the London (Queen Square) NHS Research Ethics Committee (reference 09/H0716/53) at the National Hospital for Neurology and Neurosurgery, London.

### SCA1 cohort

The patient cohort with large normal or pathogenic alleles was selected by querying the Neurogenetics Unit database at The National Hospital for Neurology and Neurosurgery, London, for individuals with SCA1 fragment sizing results ≥39 repeats. This threshold is accepted as the lower size limit of full penetrance pathogenic alleles [56]. The search identified 36 individuals, of which 35 had a diagnosis of SCA1. The other patient had a borderline SCA1 allele with 39 repeats. However, since this allele could be digested with *Sfa*NI this patient was therefore not given a diagnosis of SCA1. Concurrent tests revealed a pathogenic expansion of 69 repeats in the SCA3 allele of this patient.

### SCA1 fragment sizing

Genomic DNA was extracted from patient peripheral blood leukocytes using a FlexiGene DNA kit (QIAGEN). SCA1 alleles were amplified by PCR using GoTaq DNA polymerase (Promega) and a FAM-label introduced by the forward primer. The primers used were SCA1For_alt (FAM-5′-TGGAGGCCTATTCCACTCTG-3′) and SCA1Rev_alt (5′-TGGACGTACTGGTTCTGCTG-3′). PCR products were checked on a 4% (w/v) agarose gel and then run on an ABI 3730*xl* DNA analyser with a LIZ-500 size standard (Applied Biosystems). Fragment analysis was performed with GeneMapper software (version 4.0, Applied Biosystems). The most intense peak for each allele was selected to calculate the allele size. For alleles with 35–39 repeats, PCR products were digested with *Sfa*NI. This restriction enzyme cuts the PCR product if a CAT interruption is present and allows the distinction of large normal (interrupted) and pathogenic (uninterrupted) alleles.

### Cloning the CAG-repeat tract of SCA1 alleles

SCA1 alleles from each patient were amplified by PCR using Phusion High-Fidelity DNA Polymerase (New England Biolabs/Finnzymes) and primers which flank the CAG-repeat region and introduce restriction enzyme cleavage sites. The primers used were *ATXN1*x8 *Bam*HI Forward (5′-GGGTTGGGATCCTTCCAGTTCATTGGGTCCTC-3′) and *ATXN1*x8 *Xho*I Reverse (5′-GGTTTGCTCGAGGTGTGTGGGATCATCGTCTG-3′). The PCR reactions were purified and digested with *Bam*HI and *Xho*I restriction enzymes. The alleles were resolved on 3% (w/v) agarose-1000 gels (Invitrogen) with a resolution distance of 6.5 cm. The gels were post-stained with a counterion-dye staining solution containing 0.0025% (w/v) crystal violet and 0.0005% (w/v) methyl orange in double distilled water [57]. Individual allele PCR products were purified from the agarose gels and ligated into the pcDNA3.1(+) vector. Ligations were transformed into chemically competent Stbl3 *E. coli* (Invitrogen) (genotype F^−^ *mcr*B *mrr hsd*S20(r_B_^−^, m_B_^−^) *rec*A13 *sup*E44 *ara*-14 *gal*K2 *lac*Y1 *pro*A2 *rps*L20(Str^R^) *xyl*-5 λ^−^ *leumtl*-1). Transformants containing plasmids with inserts were propagated and the plasmids sequenced using the BigDye Terminator v3.1 Cycle Sequencing Kit (Applied Biosystems).

### Statistical analysis

A bivariate two-tailed Pearson correlation between the pathogenic allele repeat size and age at disease onset was assessed using SPSS Statistics (version 21, IBM). Analysis was only performed on patients in the cohort with a diagnosis of SCA1. Since the cloning and sequencing approach identified clones of multiple lengths due to mosaicism, the mean pathogenic allele size (≥39 repeats) across these clones was used for each patient. In addition, the data were fit to a linear model of the form “Age at Onset = A*(Pathogenic Allele Size)+b” in MATLAB (R2012b, The MathWorks) using a QR decomposition algorithm. Prediction bounds for a new observation with non-simultaneous bounds were also calculated at the 95% confidence interval.

### Construction of plasmid vectors for expression in mammalian cells

Ataxin-1cDNA encoding the C-terminal 590 aminoacids and lacking the polyQ tract (ataxinCT) was amplified and cloned into Myc-tagged pNuc vector (Invitrogen) using *Xho*I and *Not*I restriction sites. PolyQ sequences amplified from patient #1's DNA and other synthetic polyQ sequences with or without histidine interruptions (polyQH) were cloned 5′ to the ataxin-1 ataxinCT construct. Oligonucleotide annealing was carried out to create these Q/QH sequences. Since long oligos are not only expensive to make but also error prone, we adopted a sequential cloning strategy starting from a construct with 30Q. Oligos encoding the 30Q sequence had a 5′ *Sal*I sticky end and a 3′ *Xho*I sticky end. Immediately 5′ to the *Xho*I site was an *Afe*I site which is a blunt ending enzyme that recognizes and cuts in the middle of the sequence AGC GCT. After cloning the 30Q sequence into *Sal*I/*Xho*I sites in the vector, the construct was digested with *Afe*I and *Xho*I and the next set of oligos were annealed and ligated to the vector. In the second and subsequent cloning steps the first two bases on the annealed oligos (that joins the *Afe*I site on the vector) could either be AG (resulting in glutamine) or AT (histidine) hence, when required, the 31^st^ residue could be a histidine. Some oligos had histidines in the middle of the sequence. One or more of such repeats was incorporated within the next 20–30 glutamine stretch. The process was repeated a third time to create Q/QH constructs having the desired number of Q/QH tracts.

### Microscopic analysis of aggregate formation

COS cells do not express endogenous ataxin-1 yet readily express ataxin-1 upon transfection with SCA1 constructs [58] hence they were used for cell studies. COS cells were transiently transfected with expression constructs using Fugene 6 transfection reagent (Roche) and immunofluorescence was carried out as described previously [59]. Briefly, the cells were fixed 48 h after transfection using 4.0% paraformaldehyde, permeabilised with 0.2% triton X-100/PBS and probed with anti-ataxin-1 antibodies (clone N65/37 obtained from NeuroMab Facility, University of California, Davis) followed by FITC-conjugated secondary antibodies. After washing with PBS, slides were mounted using Citifluor (Agar Scientific) and analysed by confocal microscopy. Cells were examined under a Leica laser scanning confocal microscope (TCS-SP1) equipped with a DM-RXE microscope and an argon-krypton laser. Aggregate-containing cells were counted and compared with the total number of fluorescent cells to determine the percentage of aggregate-containing cells.

### Filter retardation assay for aggregate analysis

COS cells were transfected with expression constructs along with internal transfection control plasmid expressing GFP. Cells were harvested 48 hours after transfection and processed as previously described [57]. In short, cells were washed with PBS, pelleted and lysed in lysis buffer [50 mM Tris-HCl (pH 8.5), 100 mM NaCl, 1 mM EDTA, 5 mM MgCl2, 0.5% (v/v) NP 40 containing Complete Protease Inhibitors (Roche)]. Lysates were adjusted to pH 7.4 and subjected to immunoprecipitation and western blot analysis using anti-GFP polyclonal antibodies (Abcam) and peroxidase-conjugated Heavy Chain specific anti-rabbit IgG monoclonal antibodies (Sigma). Pellets containing insoluble material were resuspended in DNase buffer (20 mMTris-HCl at pH 8.0, 15 mM MgCl2) and treated with DNase I for 1 h at 37°C. Protein concentration was determined by dotMetric assay (G-Biosciences). Incubations were terminated by adding EDTA, SDS and DTT to a final concentration of 20 mM EDTA, 2% SDS and 50 mM DTT and heating at 98°C for 5 minutes. 50 µg of extracted proteins from the pellet fractions were filtered on a slot blot unit (Scie-Plas Ltd) through a cellulose acetate membrane (Sartorius, 0.2 µM pore size) pre-equilibrated with 2% SDS. After two washes with 0.1% SDS, membranes were blocked in TBS (100 mMTris-HCl at pH 7.5, 150 mMNaCl) containing 3% dried milk powder. After washing extensively with TBS, membranes were incubated with anti-ataxin-1 antibodies followed by HRP-conjugated secondary antibodies. Proteins were detected by chemiluminescence (Pierce).

### *In vitro* aggregation kinetics of uninterrupted and His-interrupted polyQ repeats

Two expanded polyQ stretches, one of uninterrupted 41 glutamines and one of the same length but interrupted with a histidine at position 22, were inserted into a pGEX-4T1 vector to be expressed as fusion proteins with Glutathione-S-transferase (GST) [14]. The resulting proteins (referred to as GSTQ41 and GSTQ41H respectively) were expressed in BL21(DE3) *E.coli* cells and affinity purified using a Glutathione Sepharose resin (GE Healthcare). ^1^H NMR samples of both constructs were prepared at a final concentration of 50 µM in 50 mM sodium phosphate buffer at pH 6.9, 3 mM DTT, 10% D_2_O. Thrombin (0.5 U/nmole of GST fusion protein) was added just before data collection. ^1^H-NMR spectra were acquired every 5 minutes on a 600 MHz Varian Inova spectrometer, at 10 and 25°C.

Thioflavin T (ThT) fluorescence assays were performed on GSTQ41 and GSTQ41H using a fluorescence plate reader (safire^2^, Tecan). Samples (25 µM) were in the same buffer used for the NMR experiments, with the addition of 20 μ MThT. 0.5 U/nmole thrombin was added in each well just before the beginning of the acquisition and fluorescence emission upon excitation at 450 nm was measured as a function of time. The reaction was carried out at 25°C. ThT fluorescence was also measured simultaneously with SLS using a home-made instrument provided with a 407 nm laser beam for the excitation of ThT and other two lasers (473 and 533 nm) for SLS measurements. Samples (50 µM) were prepared in 50 mM sodium phosphate at pH 6.9, 3 mM DTT, 300 µM ThT. The aggregation kinetics were monitored at 30°C after the addition of 0.5 U/nmole thrombin. NMR and ThT assays were repeated in triplicates on each of three independent batches of samples of polyQ41 and polyQ41H. Fibre stability of the aggregates was measured by following the light scattering intensity as a function of temperature.

## Supporting Information

Figure S1 Correlation between pathogenic allele size determined by clone sequencing and fragment sizing of clones. Mean pathogenic allele size as determined via clone sequencing was compared to diagnostic fragment sizing of clones (*n* = 100).
(PDF)

Figure S2 Correlation between pathogenic allele size determined by clone sequencing with Subjects #7, 8 and 23 highlighted. Patients with a single interrupted pathogenic clone amongst a population of uninterrupted clones are shown in red. They are indistinguishable from patients with pure uninterrupted alleles of the same size. For further details on the correlation, please refer to **Figure 1B**.
(PDF)

Table S1 Frequency with which each clone sequence was detected for each individual. The bold line indicates the pathogenic threshold of ≥39 repeats. Individual #36 (^+^) is affected with SCA3, but has a borderline interrupted expansion in SCA1. The majority of uninterrupted clones have a size above the pathogenic threshold, whilst the majority of interrupted clones lie below the pathogenic threshold.
(PDF)

Table S2 Frequency with which each clone sequence was detected for each individual, corrected for the clone depth sequenced for each patient. The bold line indicates the pathogenic threshold of ≥39 repeats. The number of clones for each patient were expressed as a percentage and these were taken into account when expressing the overall percentage of each clone within the clone population (final column).
(PDF)

Table S3 Summary of age at disease onset and allele size data for each patient. This data was used to compile the graphs in **Figure 1**.
(PDF)
