# Supplementary figures and images for: The Role of Interruptions in polyQ in the Pathology of SCA1
Source: PLoS Genet. 2013 Jul 25;9(7):e1003648. doi: 10.1371/journal.pgen.1003648 (PMC3723530; doi:10.1371/journal.pgen.1003648)

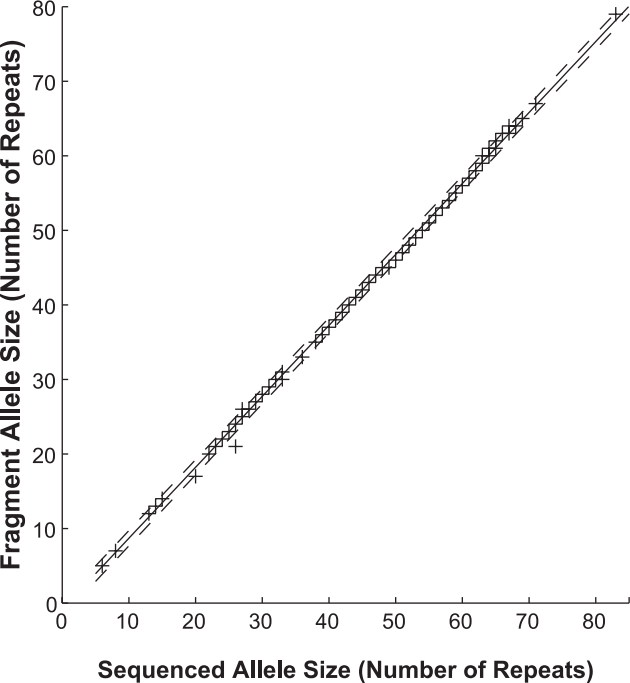

Supplement: Figure S1 — Correlation between pathogenic allele size determined by clone sequencing and fragment sizing of clones. Mean pathogenic allele size as determined via clone sequencing was compared to diagnostic fragment sizing of clones (n = 100). (PDF) [file pgen.1003648.s001.pdf]

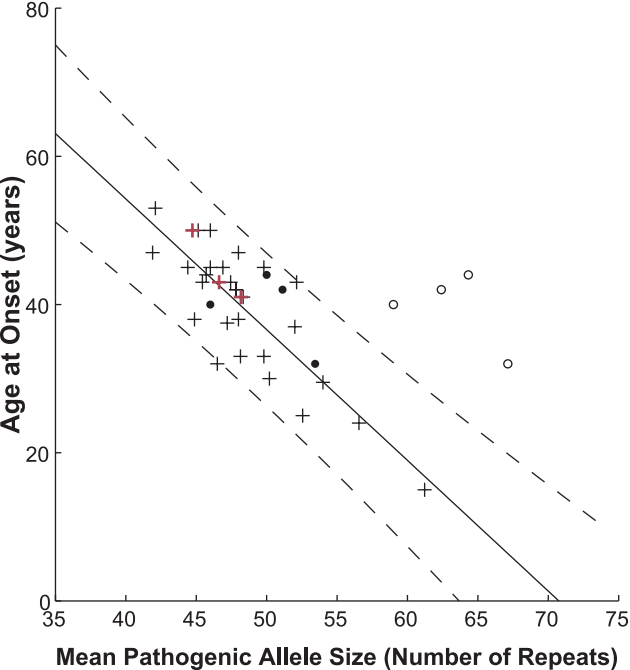

Supplement: Figure S2 — Correlation between pathogenic allele size determined by clone sequencing with Subjects #7, 8 and 23 highlighted. Patients with a single interrupted pathogenic clone amongst a population of uninterrupted clones are shown in red. They are indistinguishable from patients with pure uninterrupted alleles of the same size. For further details on the correlation, please refer to Figure 1B . (PDF) [file pgen.1003648.s002.pdf]
