# Supplementary material for: The Role of Interruptions in polyQ in the Pathology of SCA1
Source: PLoS Genet. 2013 Jul 25;9(7):e1003648. doi: 10.1371/journal.pgen.1003648 (PMC3723530; doi:10.1371/journal.pgen.1003648)
Supplement: Table S1 — Frequency with which each clone sequence was detected for each individual. The bold line indicates the pathogenic threshold of ≥39 repeats. Individual #36 (+) is affected with SCA3, but has a borderline interrupted expansion in SCA1. The majority of uninterrupted clones have a size above the pathogenic threshold, whilst the majority of interrupted clones lie below the pathogenic threshold. (PDF) [file pgen.1003648.s003.pdf]

Repeat Size	Repeat Sequence Configuration	Individual																																				Total
		1	2	3	4	5	6	7	8	9	10	11	12	13	14	15	16	17	18	19	20	21	22	23	24	25	26	27	28	29	30	31	32	33	34	35	36*	
6	$(CAG)_{6}$											1																												1
8	$(CAG)_{8}$																								1															1
13	$(CAG)_{13}$																									1														1
14	$(CAG)_{14}$		1																																				1
15	$(CAG)_{15}$						1													1																1		3
20	$(CAG)_{20}$																											1												1
22	$(CAG)_{6}(CAT)(CAG)(CAT)(CAG)_{13}$																									1														1
22	$(CAG)_{7}(CAT)(CAG)(CAT)(CAG)_{12}$																									1														1
23	$(CAG)_{7}(CAT)(CAG)(CAT)(CAG)_{13}$		1																						8														9
24	$(CAG)_{10}(CAT)(CAG)_{13}$																																		1					1
24	$(CAG)_{11}(CAT)(CAG)(CAT)(CAG)_{10}$						1																																1
25	$(CAG)_{10}(CAT)(CAG)_{14}$											2	1																					1					4
25	$(CAG)_{11}(CAT)(CAG)_{13}$																																		1					1
25	$(CAG)_{12}(CAT)(CAG)(CAT)(CAG)_{10}$						1																																1
26	$(CAG)_{26}$						1																																1
26	$(CAG)_{11}(CAT)(CAG)_{14}$											5	7																					10	1				23
26	$(CAG)_{13}(CAT)(CAG)(CAT)(CAG)_{10}$						10										7		1																				18
26	$(CAG)_{13}(CAT)(CAG)(CAT)_{2}(CAG)_{9}$						1																																1
27	$(CAG)_{10}(CAT)(CAG)_{16}$																											1												1
27	$(CAG)_{11}(CAT)(CAG)_{15}$																					1																		1
27	$(CAG)_{11}(CAT)(CAG)(CAT)(CAG)_{13}$																						1																	1
27	$(CAG)_{12}(CAT)(CAG)(CAT)(CAG)_{12}$																																					1		1
28	$(CAG)_{28}$																												1											1
28	$(CAG)_{11}(CAT)(CAG)_{16}$																					1				1		9												11
28	$(CAG)_{11}(CAT)(CAG)(CAT)(CAG)_{14}$	1													2						1						2	1	2		1					1		11
28	$(CAG)_{12}(CAT)(CAG)(CAT)(CAG)_{13}$							1	1						1						3			4			1	1	1	1								14
29	$(CAG)_{10}(CAT)(CAG)(CAT)(CAG)(CAT)(CAG)_{14}$																														1									1
29	$(CAG)_{11}(CAT)(CAG)(CAT)(CAG)_{15}$							1								1									2										1			5
29	$(CAG)_{12}(CAT)(CAG)(CAT)(CAG)_{14}$	1	2		1	12				2					2						10	12		17			12	11	12	12	12					6	3	127
29	$(CAG)_{12}(CAT)(CAG)(CAT)(CAG)(CAT)(CAG)_{12}$																									2														2
29	$(CAG)_{13}(CAT)(CAG)(CAT)(CAG)_{13}$																					1																		1
29	$(CAG)_{19}(CAT)(CAG)(CAT)(CAG)_{7}$																																						1	1
30	$(CAG)_{30}$																								1															1
30	$(CAG)_{11}(CAT)(CAG)(CAT)(CAG)_{16}$												1								1																		2
30	$(CAG)_{12}(CAT)(CAG)(CAT)(CAG)_{15}$	23	5	5	9			9	2	11				4		5								4	9		1					2		2	10		15	116
30	$(CAG)_{13}(CAT)(CAG)(CAT)(CAG)_{3}(CAT)(CAG)_{10}$										1																													1
30	$(CAG)_{13}(CAT)(CAG)(CAT)(CAG)_{14}$								15																																15
30	$(CAG)_{14}(CAT)(CAG)_{15}$								1																																1
31	$(CAG)_{31}$																					1																		1
31	$(CAG)_{12}(CAT)(CAG)(CAT)(CAG)_{16}$								2	1							1															9		10				23
31	$(CAG)_{13}(CAT)(CAG)(CAT)(CAG)_{15}$		1											1					1				3																6
31	$(CAG)_{14}(CAT)(CAG)(CAT)(CAG)_{14}$								2		1			1									2																6
32	$(CAG)_{32}$																								1															1
32	$(CAG)_{14}(CAT)(CAG)(CAT)(CAG)_{15}$		6						8		3			1		1			6	1			25	1														52
32	$(CAG)_{14}(CAT)(CAG)(CAT)(CAG)_{4}(CAT)(CAG)_{10}$																								1															1
33	$(CAG)_{33}$												1		1			1																					3
33	$(CAG)_{14}(CAT)(CAG)(CAT)(CAG)_{16}$																	12																					12
36	$(CAG)_{36}$																									1														1
38	$(CAG)_{38}$																						1																	1
38	$(CAG)_{20}(CAT)(CAG)(CAT)(CAG)_{15}$							1																																1
39	$(CAG)_{39}$																												1	1										2
39	$(CAG)_{18}(CAT)(CAG)(CAT)(CAG)_{18}$																																						1	1
39	$(CAG)_{22}(CAT)(CAG)(CAT)(CAG)_{14}$																												1											1
40	$(CAG)_{40}$	2										1																										3
40	$(CAG)_{19}(CAT)(CAG)(CAT)(CAG)_{18}$																																						1	1
40	$(CAG)_{20}(CAT)(CAG)(CAT)(CAG)_{17}$																																						4	4
41	$(CAG)_{41}$								1															1	2			2	2										8
41	$(CAG)_{20}(CAT)(CAG)(CAT)(CAG)_{18}$																																						11	11
42	$(CAG)_{42}$																									1			3	3										7
42	$(CAG)_{20}(CAT)(CAG)(CAT)(CAG)_{19}$																																						1	1
42	$(CAG)_{25}(CAT)(CAG)(CAT)(CAG)_{14}$																									1														1
43	$(CAG)_{43}$								2																2		3	1	2		2				1				13
44	$(CAG)_{44}$														1		3								1		2	1	2	2	2					1			15
45	$(CAG)_{45}$						1			1			2	2	1	1						1	1	1								2						5			18
46	$(CAG)_{46}$						1	2		6			1	1		2	1			1		1			1	1	2	1		4						1			2		28
47	$(CAG)_{47}$						1	2	3	2	4		1	2	2	3	1	1		1		1	1	2		1				1								1	1		31
47	$(CAG)_{30}(CAT)(CAG)(CAT)(CAG)_{14}$								1																																1
48	$(CAG)_{48}$						1	4		4		3	1		1	3	1					1	2	1	1	2					1								2		28
48	$(CAG)_{4}(AAG)(CAG)_{43}$									1																															1
48	$(CAG)_{12}(CAT)(CAG)(CAT)(CAG)_{33}$							1																																1
49	$(CAG)_{49}$						1	1	2			1		2		1			3				2	2	1	2														18
50	$(CAG)_{50}$								2		1						1			2			3			1					1		1						12
51	$(CAG)_{51}$							1								1					1	2								1		1						7
52	$(CAG)_{52}$					2											1			2			5								3					1		14
53	$(CAG)_{53}$																1								1							2	2	3					9
54	$(CAG)_{54}$																2															1	1	1	1				6
55	$(CAG)_{55}$										1			1			1																		3				6
56	$(CAG)_{56}$																																		1	2				3
57	$(CAG)_{57}$																1																2						3
58	$(CAG)_{58}$																																	2		1				3
59	$(CAG)_{59}$																	2															1						3
59	$(CAG)_{46}(CAT)(CAG)(CAT)(CAG)_{10}$		1																																				1
60	$(CAG)_{60}$																	3					1										1						5
61	$(CAG)_{61}$																	1																					1
62	$(CAG)_{62}$																	1																					1
62	$(CAG)_{50}(CAT)(CAG)(CAT)(CAG)_{9}$				1																																		1
63	$(CAG)_{63}$																	1																					1
63	$(CAG)_{50}(CAT)(CAG)(CAT)(CAG)_{10}$				1																																		1
64	$(CAG)_{51}(CAT)(CAG)(CAT)(CAG)_{10}$	1			1																																		2
64	$(CAG)_{52}(CAT)(CAG)(CAT)(CAG)_{9}$			1	1																																		2
65	$(CAG)_{4}(CAT)(CAG)_{6}(CAT)(CAG)_{40}(CAT)(CAG)(CAT)(CAG)_{10}$			1																																			1
65	$(CAG)_{12}(CAT)(CAG)(CAT)(CAG)_{37}(CAT)_{3}(CAG)_{10}$				1																																		1
65	$(CAG)_{52}(CAT)(CAG)(CAT)(CAG)_{10}$	6		7	2																																		15
65	$(CAG)_{53}(CAT)(CAG)(CAT)(CAG)_{9}$				1																																		1
66	$(CAG)_{53}(CAT)(CAG)(CAT)(CAG)_{10}$	3		1	1																																		5
67	$(CAG)_{67}$																	1																					1
67	$(CAG)_{54}(CAT)(CAG)(CAT)(CAG)_{10}$	1		2																																				3
68	$(CAG)_{55}(CAT)(CAG)(CAT)(CAG)_{10}$	1		2																																				3
69	$(CAG)_{56}(CAT)(CAG)(CAT)(CAG)_{10}$	1																																						1
71	$(CAG)_{58}(CAT)(CAG)(CAT)(CAG)_{10}$			1																																			1
83	$(CAG)_{70}(CAT)(CAG)(CAT)(CAG)_{10}$			1																																			1
	**Total**	40	17	21	19	18	24	18	52	19	10	15	15	15	15	14	17	22	13	12	20	19	51	52	17	19	27	23	25	18	21	20	21	21	18	15	37	800
