# Supplementary material for: The Role of Interruptions in polyQ in the Pathology of SCA1
Source: PLoS Genet. 2013 Jul 25;9(7):e1003648. doi: 10.1371/journal.pgen.1003648 (PMC3723530; doi:10.1371/journal.pgen.1003648)
Supplement: Table S2 — Frequency with which each clone sequence was detected for each individual, corrected for the clone depth sequenced for each patient. The bold line indicates the pathogenic threshold of ≥39 repeats. The number of clones for each patient were expressed as a percentage and these were taken into account when expressing the overall percentage of each clone within the clone population (final column). (PDF) [file pgen.1003648.s004.pdf]

Repeat Size	Repeat Sequence Configuration	Individual																																				Total
		1	2	3	4	5	6	7	8	9	10	11	12	13	14	15	16	17	18	19	20	21	22	23	24	25	26	27	28	29	30	31	32	33	34	35	36[+]	
6	$(CAG)_{6}$											6.7																										0.2
8	$(CAG)_{8}$																						2.0															0.1
13	$(CAG)_{13}$																							1.9														0.1
14	$(CAG)_{14}$		5.9																																			0.2
15	$(CAG)_{15}$						4.2													8.3																6.7		0.5
20	$(CAG)_{20}$																									5.3												0.1
22	$(CAG)_{6}(CAT)(CAG)(CAT)(CAG)_{13}$																							1.9														0.1
22	$(CAG)_{7}(CAT)(CAG)(CAT)(CAG)_{12}$																							1.9														0.1
23	$(CAG)_{7}(CAT)(CAG)(CAT)(CAG)_{13}$		5.9																					15.4														0.6
24	$(CAG)_{10}(CAT)(CAG)_{13}$																																4.8					0.1
24	$(CAG)_{11}(CAT)(CAG)(CAT)(CAG)_{10}$						4.2																															0.1
25	$(CAG)_{10}(CAT)(CAG)_{14}$											13.3	6.7																				4.8					0.7
25	$(CAG)_{11}(CAT)(CAG)_{13}$																																4.8					0.1
25	$(CAG)_{12}(CAT)(CAG)(CAT)(CAG)_{10}$						4.2																															0.1
26	$(CAG)_{26}$						4.2																															0.1
26	$(CAG)_{11}(CAT)(CAG)_{14}$											33.3	46.7																				47.6	4.8				3.7
26	$(CAG)_{13}(CAT)(CAG)(CAT)(CAG)_{10}$						41.7										41.2		7.7																			2.5
26	$(CAG)_{13}(CAT)(CAG)(CAT)_{2}(CAG)_{9}$						4.2																															0.1
27	$(CAG)_{10}(CAT)(CAG)_{16}$																									5.3												0.1
27	$(CAG)_{11}(CAT)(CAG)_{15}$																			8.3																		0.2
27	$(CAG)_{11}(CAT)(CAG)(CAT)(CAG)_{13}$																				5.0																	0.1
27	$(CAG)_{12}(CAT)(CAG)(CAT)(CAG)_{12}$																																			6.7		0.2
28	$(CAG)_{28}$																										3.7											0.1
28	$(CAG)_{11}(CAT)(CAG)_{16}$																			8.3				1.9		47.4												1.6
28	$(CAG)_{11}(CAT)(CAG)(CAT)(CAG)_{14}$	2.5													13.3						5.0						7.4	4.3	8.0		4.8					6.7		1.4
28	$(CAG)_{12}(CAT)(CAG)(CAT)(CAG)_{13}$							5.6	1.9						6.7						15.0			7.7			3.7	4.3	4.0	5.6								1.5
29	$(CAG)_{10}(CAT)(CAG)(CAT)(CAG)(CAT)(CAG)_{14}$																												4.0									0.1
29	$(CAG)_{11}(CAT)(CAG)(CAT)(CAG)_{15}$							5.6								7.1									11.8										5.6			0.8
29	$(CAG)_{12}(CAT)(CAG)(CAT)(CAG)_{14}$	2.5	11.8		5.3	66.7				10.5					13.3						50.0	63.2		32.7			44.4	47.8	48.0	66.7	57.1					40.0	8.1	15.8
29	$(CAG)_{12}(CAT)(CAG)(CAT)(CAG)(CAT)(CAG)_{12}$																							3.8														0.1
29	$(CAG)_{13}(CAT)(CAG)(CAT)(CAG)_{13}$																			8.3																		0.2
29	$(CAG)_{19}(CAT)(CAG)(CAT)(CAG)_{7}$																																				2.7	0.1
30	$(CAG)_{30}$																						2.0															0.1
30	$(CAG)_{11}(CAT)(CAG)(CAT)(CAG)_{16}$												6.7							8.3																		0.4
30	$(CAG)_{12}(CAT)(CAG)(CAT)(CAG)_{15}$	57.5	29.4	23.8	47.4			50.0	3.8	57.9				26.7		35.7								7.7	52.9		3.7					10.0		9.5	55.6		40.5	14.2
30	$(CAG)_{13}(CAT)(CAG)(CAT)(CAG)_{3}(CAT)(CAG)_{10}$										10.0																											0.3
30	$(CAG)_{13}(CAT)(CAG)(CAT)(CAG)_{14}$								28.8																													0.8
30	$(CAG)_{14}(CAT)(CAG)_{15}$								1.9																													0.1
31	$(CAG)_{31}$																			8.3																		0.2
31	$(CAG)_{12}(CAT)(CAG)(CAT)(CAG)_{16}$								3.8	5.3							5.9															45.0		47.6				3.0
31	$(CAG)_{13}(CAT)(CAG)(CAT)(CAG)_{15}$		5.9											6.7					7.7				5.9															0.7
31	$(CAG)_{14}(CAT)(CAG)(CAT)(CAG)_{14}$								3.8		10.0			6.7									3.9															0.7
32	$(CAG)_{32}$																						2.0															0.1
32	$(CAG)_{14}(CAT)(CAG)(CAT)(CAG)_{15}$		35.3						15.4		30.0			6.7		7.1			46.2	8.3			49.0	1.9														5.6
32	$(CAG)_{14}(CAT)(CAG)(CAT)(CAG)_{4}(CAT)(CAG)_{10}$																						2.0															0.1
33	$(CAG)_{33}$												6.7		6.7			4.5																				0.5
33	$(CAG)_{14}(CAT)(CAG)(CAT)(CAG)_{16}$																	54.5																				1.5
36	$(CAG)_{36}$																							1.9														0.1
38	$(CAG)_{38}$																				5.0																	0.1
38	$(CAG)_{20}(CAT)(CAG)(CAT)(CAG)_{15}$							5.6																														0.2
39	$(CAG)_{39}$																										3.7	4.3										0.2
39	$(CAG)_{18}(CAT)(CAG)(CAT)(CAG)_{18}$																																				2.7	0.1
39	$(CAG)_{22}(CAT)(CAG)(CAT)(CAG)_{14}$																										3.7											0.1
40	$(CAG)_{40}$	5.0										6.7																										0.3
40	$(CAG)_{19}(CAT)(CAG)(CAT)(CAG)_{18}$																																				2.7	0.1
40	$(CAG)_{20}(CAT)(CAG)(CAT)(CAG)_{17}$																																				10.8	0.3
41	$(CAG)_{41}$								1.9														2.0	3.8			7.4	8.7										0.7
41	$(CAG)_{20}(CAT)(CAG)(CAT)(CAG)_{18}$																																				29.7	0.8
42	$(CAG)_{42}$																							1.9			11.1	13.0										0.7
42	$(CAG)_{20}(CAT)(CAG)(CAT)(CAG)_{19}$																																				2.7	0.1
42	$(CAG)_{25}(CAT)(CAG)(CAT)(CAG)_{14}$																							1.9														0.1
43	$(CAG)_{43}$								3.8															3.8		15.8	3.7	8.7		11.1				4.8				1.4
44	$(CAG)_{44}$													6.7		21.4								1.9		10.5	3.7	8.7	8.0	11.1					5.6			2.2
45	$(CAG)_{45}$					5.6			1.9			13.3	13.3	6.7	6.7					8.3	5.0	5.3							8.0						27.8			2.8
46	$(CAG)_{46}$					5.6	8.3		11.5			6.7	6.7		13.3	7.1			7.7		5.0			1.9	5.9	10.5	3.7		16.0				4.8			13.3		3.6
47	$(CAG)_{47}$					5.6	8.3	16.7	3.8	21.1		6.7	13.3	13.3	20.0	7.1	5.9		7.7		5.0	5.3	3.9		5.9				4.0						5.6	6.7		4.6
47	$(CAG)_{30}(CAT)(CAG)(CAT)(CAG)_{14}$								1.9																													0.1
48	$(CAG)_{48}$					5.6	16.7		7.7		30.0	6.7		6.7	20.0	7.1					5.0	10.5	2.0	1.9	11.8					5.6						13.3		4.2
48	$(CAG)_{4}(AAG)(CAG)_{43}$									5.3																												0.1
48	$(CAG)_{12}(CAT)(CAG)(CAT)(CAG)_{33}$							5.6																														0.2
49	$(CAG)_{49}$						4.2	5.6	3.8			6.7		13.3		7.1			23.1			10.5	3.9	1.9	11.8													2.6
50	$(CAG)_{50}$								3.8		10.0						5.9			16.7			5.9			5.3					4.8		4.8					1.6
51	$(CAG)_{51}$							5.6									5.9					5.3	3.9								4.8		4.8					0.8
52	$(CAG)_{52}$					11.1											5.9			16.7			9.8								14.3					6.7		1.8
53	$(CAG)_{53}$																5.9							1.9							9.5	10.0	14.3					1.2
54	$(CAG)_{54}$																11.8														4.8	5.0	4.8	4.8				0.9
55	$(CAG)_{55}$										10.0			6.7			5.9																	14.3				1.0
56	$(CAG)_{56}$																																4.8	9.5				0.4
57	$(CAG)_{57}$																5.9															10.0						0.4
58	$(CAG)_{58}$																															10.0		4.8				0.4
59	$(CAG)_{59}$																	9.1														5.0						0.4
59	$(CAG)_{46}(CAT)(CAG)(CAT)(CAG)_{10}$		5.9																																			0.2
60	$(CAG)_{60}$																	13.6					2.0									5.0						0.6
61	$(CAG)_{61}$																	4.5																				0.1
62	$(CAG)_{62}$																	4.5																				0.1
62	$(CAG)_{50}(CAT)(CAG)(CAT)(CAG)_{9}$				5.3																																	0.1
63	$(CAG)_{63}$																	4.5																				0.1
63	$(CAG)_{50}(CAT)(CAG)(CAT)(CAG)_{10}$				5.3																																	0.1
64	$(CAG)_{51}(CAT)(CAG)(CAT)(CAG)_{10}$	2.5			5.3																																	0.2
64	$(CAG)_{52}(CAT)(CAG)(CAT)(CAG)_{9}$			4.8	5.3																																	0.3
65	$(CAG)_{4}(CAT)(CAG)_{6}(CAT)(CAG)_{40}(CAT)(CAG)(CAT)(CAG)_{10}$			4.8																																		0.1
65	$(CAG)_{12}(CAT)(CAG)(CAT)(CAG)_{37}(CAT)_{3}(CAG)_{10}$				5.3																																	0.1
65	$(CAG)_{52}(CAT)(CAG)(CAT)(CAG)_{10}$	15.0		33.3	10.5																																	1.6
65	$(CAG)_{53}(CAT)(CAG)(CAT)(CAG)_{9}$				5.3																																	0.1
66	$(CAG)_{53}(CAT)(CAG)(CAT)(CAG)_{10}$	7.5		4.8	5.3																																	0.5
67	$(CAG)_{67}$																	4.5																				0.1
67	$(CAG)_{54}(CAT)(CAG)(CAT)(CAG)_{10}$	2.5		9.5																																		0.3
68	$(CAG)_{55}(CAT)(CAG)(CAT)(CAG)_{10}$	2.5		9.5																																		0.3
69	$(CAG)_{56}(CAT)(CAG)(CAT)(CAG)_{10}$	2.5																																				0.1
71	$(CAG)_{58}(CAT)(CAG)(CAT)(CAG)_{10}$			4.8																																		0.1
83	$(CAG)_{70}(CAT)(CAG)(CAT)(CAG)_{10}$			4.8																																		0.1
	**TOTAL**	100	100	100	100	100	100	100	100	100	100	100	100	100	100	100	100	100	100	100	100	100	100	100	100	100	100	100	100	100	100	100	100	100	100	100	100	100
