# Supplementary material for: The Role of Interruptions in polyQ in the Pathology of SCA1
Source: PLoS Genet. 2013 Jul 25;9(7):e1003648. doi: 10.1371/journal.pgen.1003648 (PMC3723530; doi:10.1371/journal.pgen.1003648)
Supplement: Table S3 — Summary of age at disease onset and allele size data for each patient. This data was used to compile the graphs in Figure 1 . (PDF) [file pgen.1003648.s005.pdf]

		Repeat Size				
		Diagnostic Sizing		Mean Sequenced		
Patient	Age at Onset (yrs)	Pathogenic	Normal	Pathogenic	Longest CAG Stretch	Normal
1	42	62	28	62	51	30
2	40	64	33	59	46	29
3	32	62	28	67	53	30
4	44	60	30	64	50	30
5	41	44	27	48	48	29
6	43	45	24	47	47	25
7	41	44	28	48	46	30
8	43	43	28	47	46	31
9	37.5	45	26	47	46	30
10	33	51	32	50	50	31
11	44	42	24	46	46	23
12	50	43	24	46	46	27
13	47	43	30	48	48	31
14	45	44	27	47	47	29
15	45	41	28	46	46	30
16	25	50	24	53	53	27
17	15	58	31	61	61	33
18	38	45	30	48	48	31
19	45	47	30	50	50	27
20	32	43	27	47	47	29
21	33	44	27	48	48	29
22	30	47	30	50	50	31
23	50	45	23	45	43	27
24	42	45	28	48	48	30
25	38	41	26	45	45	27
26	47	39	27	42	40	29
27	53	39	27	42	42	29
28	43	42	27	45	45	29
29	45	40	27	44	44	29
30	43	50	28	52	52	29
31	24	55	30	57	57	31
32	37	52	25	52	52	26
33	29.5	53	29	54	54	30
34	50	42	28	45	45	30
35	42	43	27	48	48	27
